# ICTV Virus Taxonomy Profile: *Picornaviridae*

**DOI:** 10.1099/jgv.0.000911

**Published:** 2017-09-08

**Authors:** R. Zell, E. Delwart, A. E. Gorbalenya, T. Hovi, A. M. Q. King, N. J. Knowles, A. M. Lindberg, M. A. Pallansch, A. C. Palmenberg, G. Reuter, P. Simmonds, T. Skern, G. Stanway, T. Yamashita, ICTV Report Consortium

**Affiliations:** ^1^​ Department of Virology and Antiviral Therapy, Jena University Hospital, Friedrich Schiller University, Jena, Germany; ^2^​ Department of Laboratory Medicine, Blood System Research Institute, University of California, San Francisco, CA, USA; ^3^​ Department of Medical Microbiology, Leiden University Medical Center, Leiden, The Netherlands; ^4^​ National Institute for Health and Welfare (THL), Helsinki, Finland; ^5^​ The Pirbright Institute, Woking, Surrey, UK; ^6^​ Department of Chemistry and Biomedical Sciences, Linnaeus University, Kalmar, Sweden; ^7^​ Division of Viral Diseases, Centers for Disease Control and Prevention (CDC), Atlanta, GA, USA; ^8^​ Department of Biochemistry, Institute for Molecular Virology, Madison, WI, USA; ^9^​ Department of Medical Microbiology and Immunology, University of Pécs, Pécs, Hungary; ^10^​ Nuffield Department of Medicine, University of Oxford, Oxford, UK; ^11^​ Max F. Perutz Laboratories, Medical University of Vienna, Vienna, Austria; ^12^​ Department of Biological Sciences, University of Essex, Colchester, UK; ^13^​ Department of Food Science and Nutrition, Faculty of Health and Nutrition, Shubun University, Aichi, Japan

**Keywords:** *Picornaviridae*, ICTV, taxonomy, poliovirus, foot-and-mouth disease virus, rhinovirus, enterovirus

## Abstract

The family *Picornaviridae* comprises small non-enveloped viruses with RNA genomes of 6.7 to 10.1 kb, and contains >30 genera and >75 species. Most of the known picornaviruses infect mammals and birds, but some have also been detected in reptiles, amphibians and fish. Many picornaviruses are important human and veterinary pathogens and may cause diseases of the central nervous system, heart, liver, skin, gastrointestinal tract or upper respiratory tract. Most picornaviruses are transmitted by the faecal–oral or respiratory routes. This is a summary of the International Committee on Taxonomy of Viruses (ICTV) Report on the taxonomy of the *Picornaviridae*, which is available at www.ictv.global/report/picornaviridae.

## Abbreviation

IRES, internal ribosome entry site.

## Virion

**Fig. 1.**
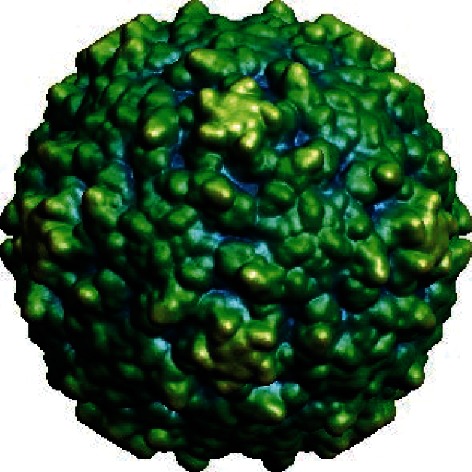
Surface view of the poliovirus 1 (*Enterovirus C*) virion (1HXS). Reproduced from VIPERdb (http://viperdb.scripps.edu) [7].

Non-enveloped, icosahedral capsids with *T*=1 (pseudo *T*=3) symmetry (Table 1; small stellated dodecahedron; Fig. 1) are composed of 60 identical protomers, each comprising 4 (1A and paralogous 1B, 1C and 1D) capsid proteins, or 3 if 1AB remains uncleaved. The mature capsid proteins 1B, 1C and 1D, and the uncleaved 1AB possess a core structure of an eight-stranded ‘β-barrel’, also known as a ‘jelly roll’ [1].

**Table 1.** Characteristics of the family *Picornaviridae*

Typical member:	poliovirus 1 Mahoney (V01149), species *Enterovirus C,* genus *Enterovirus*
Virion	Non-enveloped, 30–32 nm virions comprising 60 protomers
Genome	6.7–10.1 kb of positive-sense, non-segmented RNA with a poly(A) tail
Replication	RNA synthesis occurs in reorganized cytoplasmic replication organelles containing non-structural proteins derived from the 2BC-P3 region of the encoded polyprotein; RNA structures at the 5′ and 3′ ends of the genome direct initiation of RNA synthesis and uridylated 3B serves as the primer for synthesis of both RNA strands
Translation	Directly from genomic RNA containing an internal ribosome entry site (IRES)
Host range	Mammals, birds, reptiles, amphibians and bony fishes
Taxonomy	Member of the order *Picornavirales*; >30 genera containing >75 species

## Genome

**Fig. 2.**
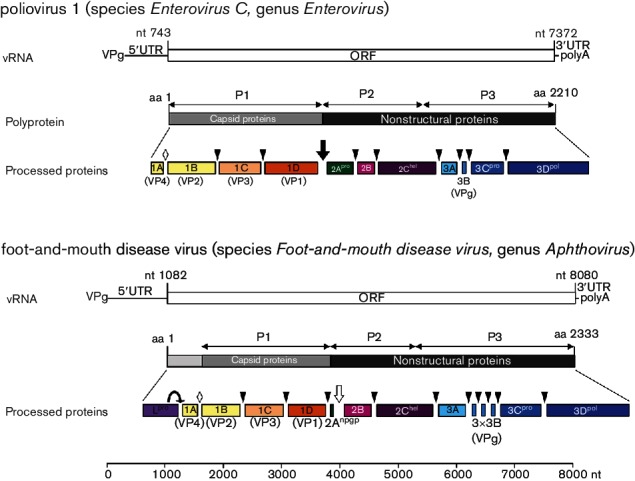
Genome organization and expression of enteroviruses and aphthoviruses. Viral RNA (vRNA) is polyadenylated and covalently linked to a virus-encoded protein (3B) at its 5′ end. Cleavages facilitated by 2A^pro^ of the enteroviruses (black arrow) or by an NPG↓P motif at the C-terminus of 2A of foot-and-mouth disease virus (white arrow) release the P1 and P1-2A proteins, respectively. The leader proteinase L^pro^ releases itself from the polyprotein by cleavage at its own C-terminus. P2 and P3 polypeptides are precursors of the nonstructural proteins necessary for genome replication. Further polyprotein processing is mediated by 3C^pro^ (cleavage sites indicated by arrow heads). Processing of 1AB, the precursor of 1A and 1B, is thought to be autocatalytic and occurs in empty capsids or at virion maturation (white diamond).

The genomic RNA, ranging in size from 6.7 kb to 10.1 kb [2], commonly contains a single large open reading frame (ORF) coding for a polyprotein (Fig. 2) [1]. A typical picornavirus genome encodes three or four capsid proteins and at least seven non-structural proteins. The ORF is flanked by untranslated regions of variable lengths. The 5′ untranslated region (UTR) (494–1451 nt) and the 3′ UTR (25–795 nt) contain RNA structures [e.g. the internal ribosome entry site (IRES)] that are essential for genome function. A long poly(C) tract is found in the 5′ UTR of foot-and-mouth disease virus (genus *Aphthovirus*) and encephalomyocarditis virus (genus *Cardiovirus*), and shorter ones may be present in porcine teschovirus 1 (genus *Teschovirus*) and equine rhinitis A virus (genus *Aphthovirus*). Picornaviruses exhibit a modular genome organization. The IRES element and the 2A genome region may have been exchanged between the genera. There may be more than one protein produced from the L or 2A genome regions, as well as multiple copies of 3B in the picornavirus genome. The genome of viruses of the genus *Dicipivirus* includes an additional IRES inserted downstream of 1D, leading to a bicistronic genome organization.

## Replication

The replication cycle starts following translational initiation on the genomic RNA, in a cap-independent manner directed by one or two IRESs of one of five types [3]. A polyprotein is synthesized that is co- and post-translationally cleaved to capsid proteins (derived from the P1 region of the genome; ORF1 of viruses in the genus *Dicipivirus*) and non-structural proteins (from the P2 and P3 regions; ORF2 of viruses in the genus *Dicipivirus*) by cognate viral proteinases (Fig. 2). These are 3C proteinase (3C^pro^) encoded by all picornaviruses, 2A^pro^ of viruses in the genus *Enterovirus* and possibly *Sapelovirus* and *Harkavirus* (both are chymotrypsin-like cysteine proteinases), and papain-like L^pro^ of viruses in the genera *Aphthovirus*, *Erbovirus* and possibly *Mosavirus*. There are a few other forms of 2A, one of which is found in many picornaviruses and contains a NPG↓P motif that mediates in *cis* co-translational termination–reinitiation of RNA translation. Replication of viral RNA occurs in complexes associated with cytoplasmic membranes [4, 5] that contain most of the functional proteins and their precursors. Uridylated 3B protein (VPg-pUpU_OH_) serves as the primer for both positive- and negative-strand RNA synthesis [6]. Where it occurs, cleavage of 1AB (VP0) accompanies virion morphogenesis.

## Taxonomy

The family *Picornaviridae* includes >30 genera and >75 species. Members of species share (i) a significant degree of amino acid identity of the P1, 2C, 3C and 3D proteins; (ii) monophyly in phylogenetic trees; and (iii) essentially identical genome maps. Members of a genus normally share (i) >33 % amino acid identity in P1 and >36 % amino acid identity in the non-structural proteins 2C+3 CD; (ii) monophyly in phylogenetic trees; and (iii) homologous L (if present), 2B, 3A and 3B proteins. Other related viruses detected in various hosts are likely to belong to additional genera and species.

## Resources

Full ICTV Online (10th) Report: www.ictv.global/report/picornaviridae. Sequence compilations: http://www.picornaviridae.com/, http://www.picornastudygroup.com/.
